# Anemhupehins A–C, Podocarpane Diterpenoids from *Anemone hupehensis*

**DOI:** 10.1007/s13659-017-0146-6

**Published:** 2017-12-11

**Authors:** Xing Yu, Kai-Ting Duan, Zhen-Xiong Wang, He-Ping Chen, Xiao-Qing Gan, Rong Huang, Zheng-Hui Li, Tao Feng, Ji-Kai Liu

**Affiliations:** School of Pharmaceutical Sciences, South-Central University for Nationalities, Wuhan, 430074 China

**Keywords:** *Anemone hupehensis*, Podocarpane diterpenoids, Cytotoxicity

## Abstract

**Abstract:**

Three new podocarpane diterpenoids, namely anemhupehins A–C (**1**–**3**), together with four known analogues (**4**–**7**), have been isolated from aerial parts of *Anemone hupehensis*. Their structures were characterized based on extensive spectroscopic data. Compounds **1** and **4** showed certain cytotoxicities against human cancer cell lines.

**Graphical Abstract:**

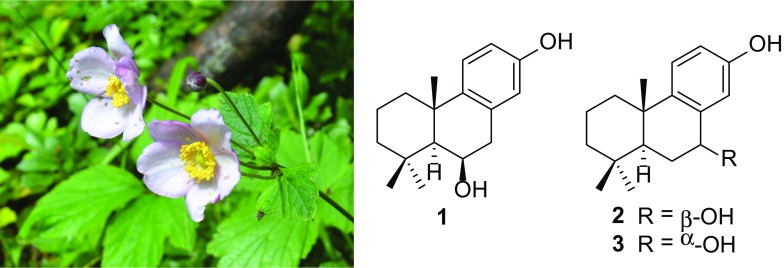

**Electronic supplementary material:**

The online version of this article (10.1007/s13659-017-0146-6) contains supplementary material, which is available to authorized users.

## Introduction

Podocarpane diterpenoids usually possessed a backbone of seventeen carbons arranged in a tricyclohexane system close to abietane and pimarane [1–8]. They do not occur extensively in nature but are present in several genera, such as *Azadirachta* [1–5], *Humirianthera* [6], *Micrandropsis* [7], and *Podocarpus* [8]. Previous studies have disclosed a few biologically active podocarpane molecules, such as 13-hydroxy-8,11,13-podocarpatriene-19-oic acid, a highly fungistatic agent [9, 10], and 7-oxo-13-hydroxy-14-amino-8,11,13-podocarpatriene, a potent 5-lipoxygenase inhibitor [11].

**Fig. 1:**
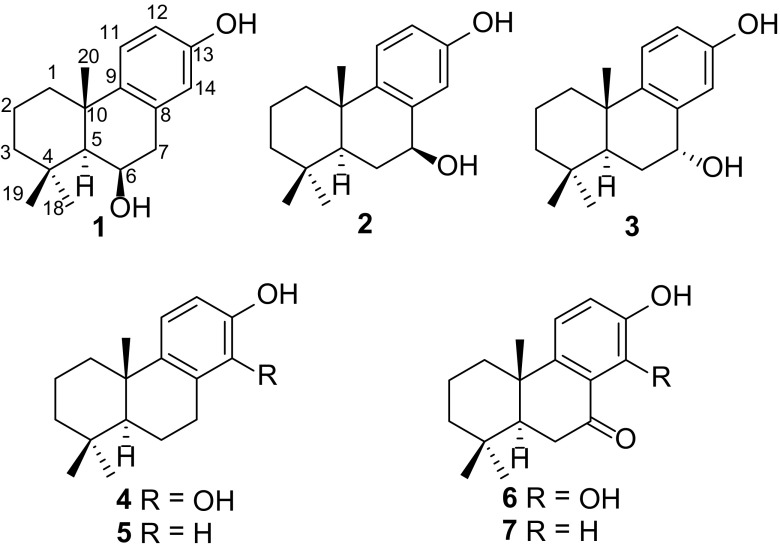
Structures of compounds **1**–**7**

*Anemone hupehensis* is a flowering herbaceous perennial in the Ranunculaceae family. It is about one meter high and native in central China. In previous chemical studies on this plant, a few triterpenoids and their saponins were reported [12–15]. In our continuous searching for new and bioactive natural products, we investigated the chemical constituents in aerial parts of *A. hupehensis*, which resulted in the isolation of three new podocarpane diterpenoids, anemhupehins A–C (**1**–**3**), as well as four known ones **4**–**7** (Fig. 1). Compound **4** was isolated as a new natural product for the first time. Their structures were established by extensive spectroscopic methods. Compounds **1** and **4**–**7** were evaluated for their cytotoxicities against five human cancer cell lines. We report here the isolation, structure elucidation, and cytotoxicities of these isolates.

## Results and Discussion

**Fig. 2:**
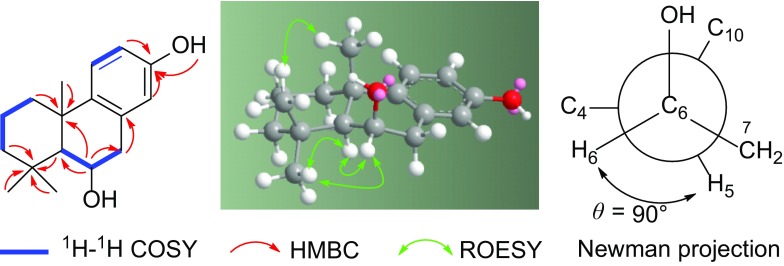
Key 2D NMR correlations of **1** and stereoconfiguration analyses

Compound **1** was isolated as a colorless oil. The HRESIMS data at *m/z* 283.1668 [M + Na]^+^ (calcd for C_17_H_24_O_2_Na: 283.1669) indicated a molecular formula C_17_H_24_O_2_, requiring six degrees of unsaturation. The IR absorption bands at 3438, 1608, and 1480 cm^−1^ were attributable to hydroxy and phenyl groups. In the ^1^H NMR spectrum (Table 1), three singlets from at *δ*_H_ 1.04 (3H), 1.27 (3H), and 1.54 (3H) were readily identified signals for three methyls, while three signals at *δ*_H_ 6.49 (1H, d, *J* = 2.8 Hz, H-14), 6.67 (1H, dd, *J* = 8.6, 2.8 Hz, H-12), and 7.17 (1H, d, *J* = 8.6 Hz, H-11) displayed a 1,3,4-trisubstituted aromatic ring. The ^13^C NMR data, with the aid of DEPT and HSQC spectra, revealed 17 carbon resonances ascribable for three methyl, four methylene, five methine, and five quaternary carbons (Table 1). Analyses of ^1^H–^1^H COSY data disclosed three spin-coupling systems shown in Fig. 2. These data, together with preliminary analyses of HMBC correlations, suggested that **1** possessed a podocarpane skeleton. A phenolic hydroxy group at *δ*_H_ 5.22 (1H, s) was deduced to be placed at C-13 by analyses of HMBC correlations and NMR data. In addition, another hydroxy group was suggested to be at C-6, as revealed by HMBC correlations from *δ*_H_ 4.67 (1H, br d, *J* = 5.0 Hz, H-6) to *δ*_C_ 53.4 (d, C-5), 41.3 (t, C-7), 34.3 (s, C-4), and 37.2 (s, C-10), as well as ^1^H–^1^H COSY cross peaks of H-6/*δ*_H_ 1.40 (1H, br s, H-5) and H-6/*δ*_H_ 3.10 and 2.85 (2H, H_2_-7). These data elucidated the structure of **1** to be 6,13-dihydroxy-8,11,13-podocarpatriene. An ROESY experiment revealed the relative configuration of **1** (Fig. 2), in which the cross peak of H-19/H-20 indicated that CH_3_-19 and CH_3_-20 were in the same side (*β* orientation), while the cross peaks of H-18/H-5, H-5/H-6, and H-6/H-18 indicated that H-6 should be *α* oriented. As established by the ROESY data, the energy minimized 3D structure of **1** revealed stereoconfiguration of **1**. A Newman projection of C-5 and C-6 indicated the dihedral angle between H-5 and H-6 to be close to *θ* = 90° (Fig. 2), which resulted in the coupling constants of *J*_5,6_ = 0 Hz, that is why H-5 presented as a singlet in the ^1^H NMR spectrum. All these data elucidated the structure of **1** to be 6*β*,13-dihydroxy-8,11,13-podocarpatriene, named anemhupehin A.

**Table 1:** ^1^H and ^13^C NMR data for compounds **1**–**3** (*δ* in ppm, *J* in Hz)^a^

No.	**1**		**2**		**3**	
	*δ*_H_	*δ*_C_	*δ*_H_	*δ*_C_	*δ*_H_	*δ*_C_
1	2.15 (1H, br d, 12.6) 1.36 (1H, m)	42.4 (t)	2.21 (1H, br d, 12.7) 1.31 (1H, m)	39.1 (t)	2.21 (1H, br d, 12.6) 1.31 (1H, m)	38.7 (t)
2	1.83 (1H, m) 1.60 (1H, m)	19.7 (t)	1.72 (1H, m) 1.58 (1H, m)	19.3 (t)	1.72 (1H, m) 1.58 (1H, m)	19.4 (t)
3	1.46 (1H, m) 1.24 (1H, m)	43.2 (t)	1.48 (1H, m) 1.21 (1H, m)	41.4 (t)	1.48 (1H, m) 1.21 (1H, m)	41.6 (t)
4		34.3 (s)		33.3 (s)		33.1 (s)
5	1.40 (1H, br s)	53.4 (d)	1.35 (1H, dd, 13.1, 3.8)	49.4 (d)	1.60 (1H, m)	44.9 (d)
6	4.67 (1H, br d, 5.0)	66.2 (d)	2.24 (1H, m) 1.64 (1H, m)	30.3 (t)	1.98 (2H, m)	28.7 (t)
7	3.10 (1H, dd, 17.4, 5.0) 2.85 (1H, d, 17.4)	41.3 (t)	4.75 (1H, m)	71.4 (d)	4.76 (1H, m)	68.4 (d)
8		133.1 (s)		139.5 (s)		137.6 (s)
9		141.3 (s)		142.5 (s)		142.6 (s)
10		37.2 (s)		38.1 (s)		37.7 (s)
11	7.17 (1H, d, 8.6)	126.7 (d)	7.10 (1H, d, 8.7)	126.1 (d)	7.14 (1H, d, 8.4)	126.1 (d)
12	6.67 (1H, dd, 8.6, 2.8)	113.9 (d)	6.72 (1H, dd, 8.7, 2.7)	115.1 (d)	6.74 (1H, dd, 8.4, 2.6)	116.0 (d)
13		153.3 (s)		153.7 (s)		153.7 (s)
14	6.49 (1H, d, 2.8)	115.5 (d)	6.99 (1H, d, 2.7)	113.3 (d)	6.82 (1H, d, 2.6)	116.0 (d)
18	1.04 (3H, s)	33.9 (q)	0.95 (3H, s)	33.2 (q)	0.97 (3H, s)	33.3 (q)
19	1.27 (3H, s)	23.9 (q)	0.92 (3H, s)	21.7 (q)	0.92 (3H, s)	21.7 (q)
20	1.54 (3H, s)	27.3 (q)	1.22 (3H, s)	25.6 (q)	1.10 (3H, s)	24.2 (q)
OH	5.22 (1H, br s)		4.96 (1H, br s)		5.37 (1H, br s)	

The assignments were based on DEPT, ^1^H-^1^H COSY, HSQC, and HMBC experiments

^a^Data (*δ*) were measured in CDCl_3_

Compound **2** was isolated as a colorless oil. The HRESIMS established the molecular formula to be C_17_H_24_O_2_ on the basis of a molecular ion peak at *m/z* 259.1704 (calcd for C_17_H_23_O_2_: 259.1704 [M − H]^−^). IR absorption bands (3432, 1612, 1481 cm^−1^) also indicated the presence of hydroxy and phenyl functions. The ^13^C NMR and DEPT disclosed 17 carbon resonances including three methyl, four methylene, five methine, and five quaternary carbons (Table 1). These data showed similar patterns to those of **1**. The ^1^H–^1^H COSY cross peak between *δ*_H_ 4.75 (1H, m, H-7) and 2.24/1.64 (each 1H, m, H-6), as well as HMBC correlations from H-7 to *δ*_C_ 139.5 (s, C-8) and 30.3 (t, C-6) suggested that a hydroxy should be placed at C-7 in **2**, rather than at C-6 in **1**. Detailed analyses of 2D NMR data indicated that the other parts of **2** were the same to those of **1**. In the ROESY spectrum, cross peaks of H-5/H-7 suggested that OH-7 should be *β* oriented. Therefore, compound **2** was elucidated as 7*β*,13-dihydroxy-8,11,13-podocarpatriene, named anemhupehin B.

Compound **3** was initially isolated as a mixture with **2**. A further isolation of this mixture only obtained pure compound **2**, while the amount of compound **3** is too less to obtain the clear NMR spectra. However, careful analyses of MS data of **3** and NMR spectra of the mixture (see Supporting Information), with the aid of NMR spectrum of **2**, can unambiguously elucidate the structure of **3**. The HRESIMS of **3** displayed a molecular ion peak at *m/z* 259.1704 (calcd for C_17_H_23_O_2_: 259.1704 [M − H]^−^), revealed the molecular formula C_17_H_24_O_2_, the same to that of **2**. The ^13^C NMR data also showed good agreements with those of **2**. Analyses of 1D and 2D NMR data suggested that compound **3** should have the same planar structure to that of **1**. However, the ^13^C NMR shift of C-7 at *δ*_C_ 68.4 in **3** was significantly different from that in **2**, indicating that compound **3** might be an epimer of **2**. In the ROESY spectrum of the mixture, the cross peaks of 19/H-20 and H-18/H-5 suggested that C-5 and C-10 possessed the same configurations to those in compounds **2**. Therefore, compound **3** was determined to be 7-epimer of **2**. The 3D structure analyses of **3** and **2** also supported that OH-7 in **3** should be *α* oriented. Therefore, compound **3** was elucidated as 7*α*,13-dihydroxy-8,11,13-podocarpatriene, named anemhupehin C.

Four known analogues were identified as 13,14-dihydroxy-8,11,13-podocarpatriene (**4**) [16], 13-hydroxy-8,11,13-podocarpatriene (**5**) [17], 13,14-dihydroxy-7-oxo-8,11,13-podocarpatriene (**6**) [17], and 13-hydroxy-7-oxo-8,11,13-podocarpatriene (**7**) [18]. Compound **4** was isolated as a natural product for the first time.

Compounds **1** and **4–7** were evaluated for their cytotoxicities to five human cancer cell lines. As a result, compounds **1** and **4** showed moderate activities as shown in Table 2. The other compounds were inactive to cancer cell lines (IC_50_ > 40 *μ*M).

**Table 2:** Cytotoxicities of compounds **1** and **4** (IC_50_, *μ*M)

Entry	HL-60	SMMC-7721	A-549	MCF-7	SW480
**1**	> 40	33.8	> 40	28.8	13.2
**4**	16.8	22.7	> 40	12.2	14.1
Cisplatin	4.4	12.4	16.4	17.1	12.8

## Experimental

### General Experimental Procedures

Optical rotations were measured on a Jasco-P-1020 polarimeter. IR spectra were obtained using a Bruker Tensor 27 FT-IR spectrometer with KBr pellets. NMR spectra were acquired with a Bruker DRX-600 with tetramethylsilane (TMS) used as an internal standard. HRESIMS were recorded on an API QSTAR Pulsar spectrometer. Silica gel (200–300 mesh), Sephadex LH-20 and RP-18 gel (20–45 *µ*m) were used for column chromatography (CC). Medium pressure liquid chromatography (MPLC) was performed on a Biotage system. Preparative high performance liquid chromatography (prep-HPLC) was performed on an Agilent 1260 liquid chromatography system equipped with Zorbax SB-C18 columns (5 μm, 9.4 mm × 150 mm or 21.2 mm × 150 mm) and a DAD detector. Fractions were monitored by TLC and spots were visualized by heating silica gel plates immersed in H_2_SO_4_ in EtOH, in combination with the Agilent 1200 series HPLC system (Eclipse XDB-C18 column, 5 μm, 4.6 × 150 mm).

### Plant Material

Aerial parts of *A. hupehensis* were collected from ShenLongJia of Hubei province, central China in September 2016 and identified by Prof. Ming-Qing Pan of Wuhan University. A specimen (No. COP-HF20160912.4) was deposited at South-Central University for Nationalities.

### Extraction and Isolation

The air dried sample of *A. hupehensis* (8 kg) were extracted with methanol (24 h × 3) to afford an extract. The extract was partitioned with water and EtOAc (1:1). The extract of EtOAc lay (202 g) was subjected to silica gel CC using CHCl_3_-MeOH (from 1:0 to 0:1) to give eight fractions (A–H). Fraction C (23.8 g) was separated by MPLC with gradient mixture of MeOH and H_2_O (20:80–100:0, v/v) to afford ten sub-fractions (C1–C10). Fraction C2 (500 mg) was isolated by silica gel CC using petroleum ether/Me_2_CO (6:1) to afford several subfractions, and compound **6** (13 mg) crystallized from one subfraction. Fraction C4 (280 mg) was purified by Sephadex LH-20 (MeOH) to give compounds **1** (7.2 mg) and **4** (8.6 mg). Compounds **2** and **3** was initially isolated as a mixture (2.8 mg) as obtained from fraction C5 (120 mg) by silica gel CC, and was re-purified by HPLC (MeCN:H_2_O (v/v) from 2:8 to 4:6 in 25 min) to afford pure compounds **2** (1.1 mg) and **3** (less than 0.5 mg). Fraction C6 (300 mg) was isolated by Sephadex LH-20 (MeOH) and then purified by HPLC (MeCN:H_2_O (v/v) from 3:7 to 4:6 in 25 min) to afford **5** (6.8 mg) and **7** (3.4 mg).

#### Anemhupehin A (**1**)

Colorless oil, $\left[ \alpha \right]_{\text{D}}^{ 2 2} + 1 7. 6$ (*c* 0.18 MeOH); IR (KBr) ν_max_ 3443, 3438, 2928, 1624, 1608, 1480, 1367, 1201, 862 cm^−1^; for ^1^H (600 MHz) and ^13^C NMR (150 MHz) data (CDCl_3_), see Table 1; HRESIMS: *m/z* 283.1668 (calcd for C_17_H_24_O_2_Na, [M + Na]^+^, 283.1669).

#### Anemhupehin B (**2**)

Colorless oil, $\left[ \alpha \right]_{\text{D}}^{ 2 2} + 1 2. 7$ (*c* 0.08 MeOH); IR (KBr) ν_max_ 3441, 3432, 2926, 1612, 1481, 1381, 1211, 882 cm^−1^; for ^1^H (600 MHz) and ^13^C NMR (150 MHz) data (CDCl_3_), see Table 1; HRESIMS: *m/z* 259.1704 (calcd for C_17_H_23_O_2_, [M − H]^−^, 259.1704).

#### Anemhupehin C (**3**)

Colorless oil, $\left[ \alpha \right]_{\text{D}}^{ 2 2} + 8. 7$ (*c* 0.02 MeOH); for ^1^H (600 MHz) and ^13^C NMR (150 MHz) data (CDCl_3_), see Table 1; HRESIMS: *m/z* 259.1704 (calcd for C_17_H_23_O_2_, [M − H]^−^, 259.1704).

### Cytotoxicity Assay

Human myeloid leukemia HL-60, hepatocellular carcinoma SMMC-7721, lung cancer A-549 cells, breast cancer MCF-7 and colon cancer SW480 cell lines were used in the cytoxic assay. All cell lines were cultured in RPMI-1640 or DMEM medium (Hyclone, USA), supplemented with 10% fetal bovine serum (Hyclone, USA) in 5% CO_2_ at 37 °C. The cytotoxicity assay was performed according to the MTT (3-(4,5-dimethylthiazol-2-yl)-2,5-diphenyl tetrazolium bromide) method in 96-well microplates [19]. Cisplatin was used as a positive control.

## Electronic supplementary material

Below is the link to the electronic supplementary material.

Supplementary material 1 (PDF 1409 kb)
