# Supplementary material for: Anemhupehins A–C, Podocarpane Diterpenoids from Anemone hupehensis
Source: Nat Prod Bioprospect. 2017 Dec 11;8(1):31–5. doi: 10.1007/s13659-017-0146-6 (PMC5803142; doi:10.1007/s13659-017-0146-6)

Supporting information for

Anemhupehins A–C, Podocarpane Diterpenoids from *Anemone hupehensis*

Xing Yu, Kai-Ting Duan, Zhen-Xiong Wang, He-Ping Chen, Xiao-Qing Gan, Rong Huang, Zheng-Hui Li, Tao Feng,* Ji -Kai Liu*

School of Pharmaceutical Sciences, South-Central University for Nationalities, Wuhan, 430074, China

** Corresponding author: tfeng@mail.scuec.edu.cn; jkliu@mail.kib.ac.cn*

List of Supporting Information

Figure S1-S7: NMR Spectra and HRESIMS of Anemhupehin A (**1**)

Figure S8-S14: NMR Spectra and HRESIMS of Anemhupehin B (**2**)

Figure S15-S21: NMR Spectra of the mixture Anemhupehins B + C (**2** + **3**) and HRESIMS of Anemhupehin C (**3**)

Figure S1. ^{1}H NMR spectrum of anemhupehin A (**1**)

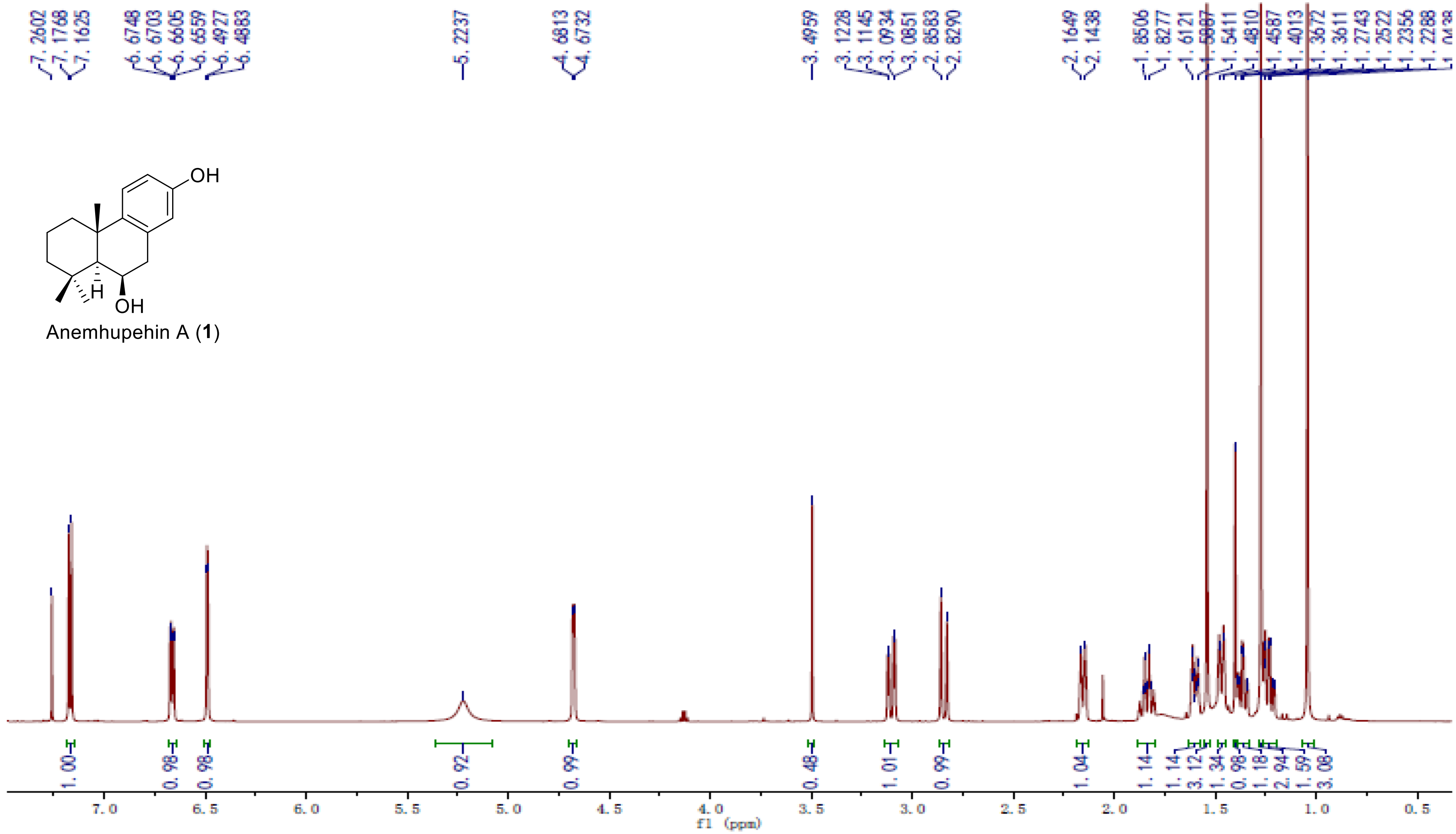

Figure S2. ^{13}C and DEPT NMR spectra of anemhupehin A (**1**)

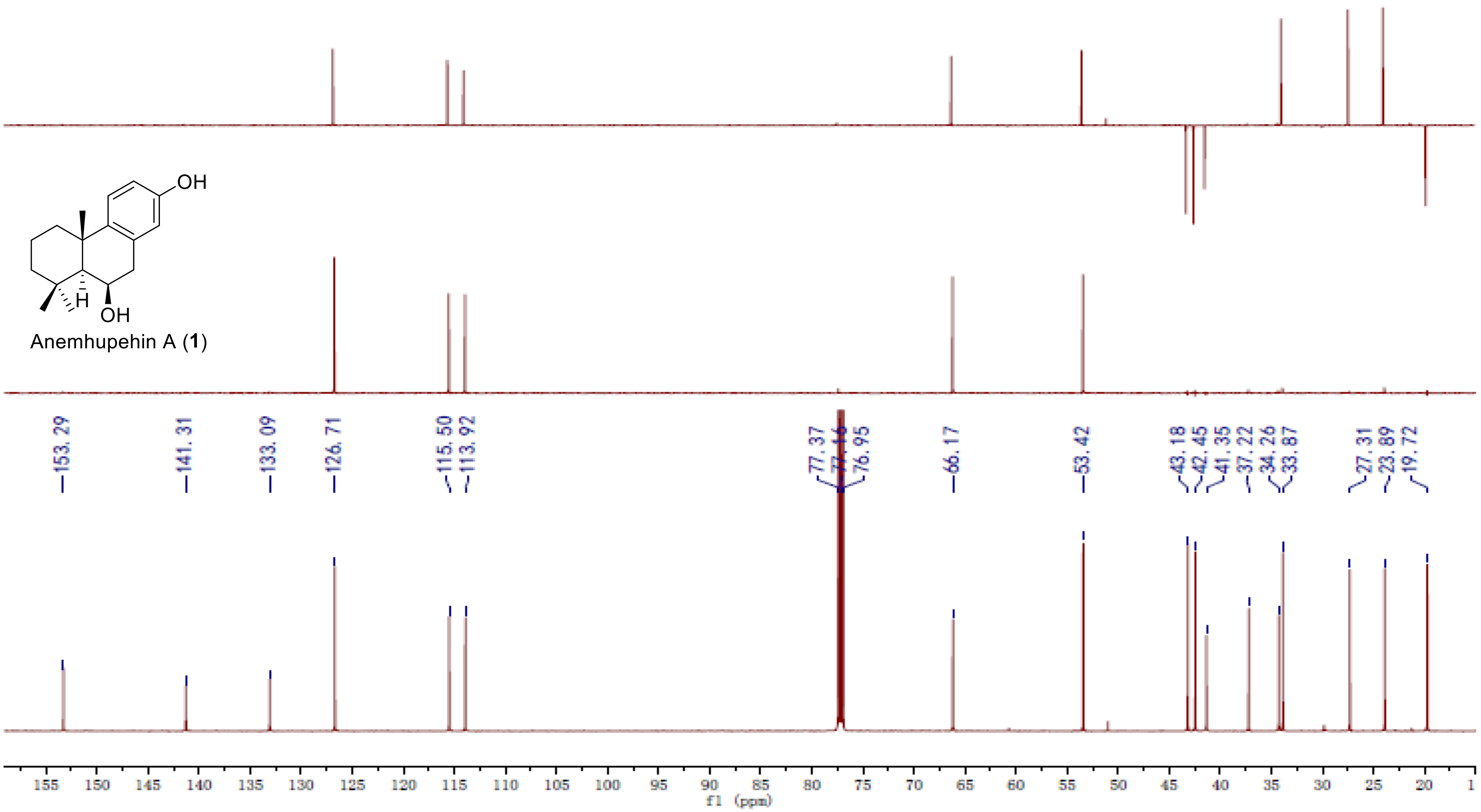

Figure S3. HSQC spectrum of anemhupehin A (**1**)

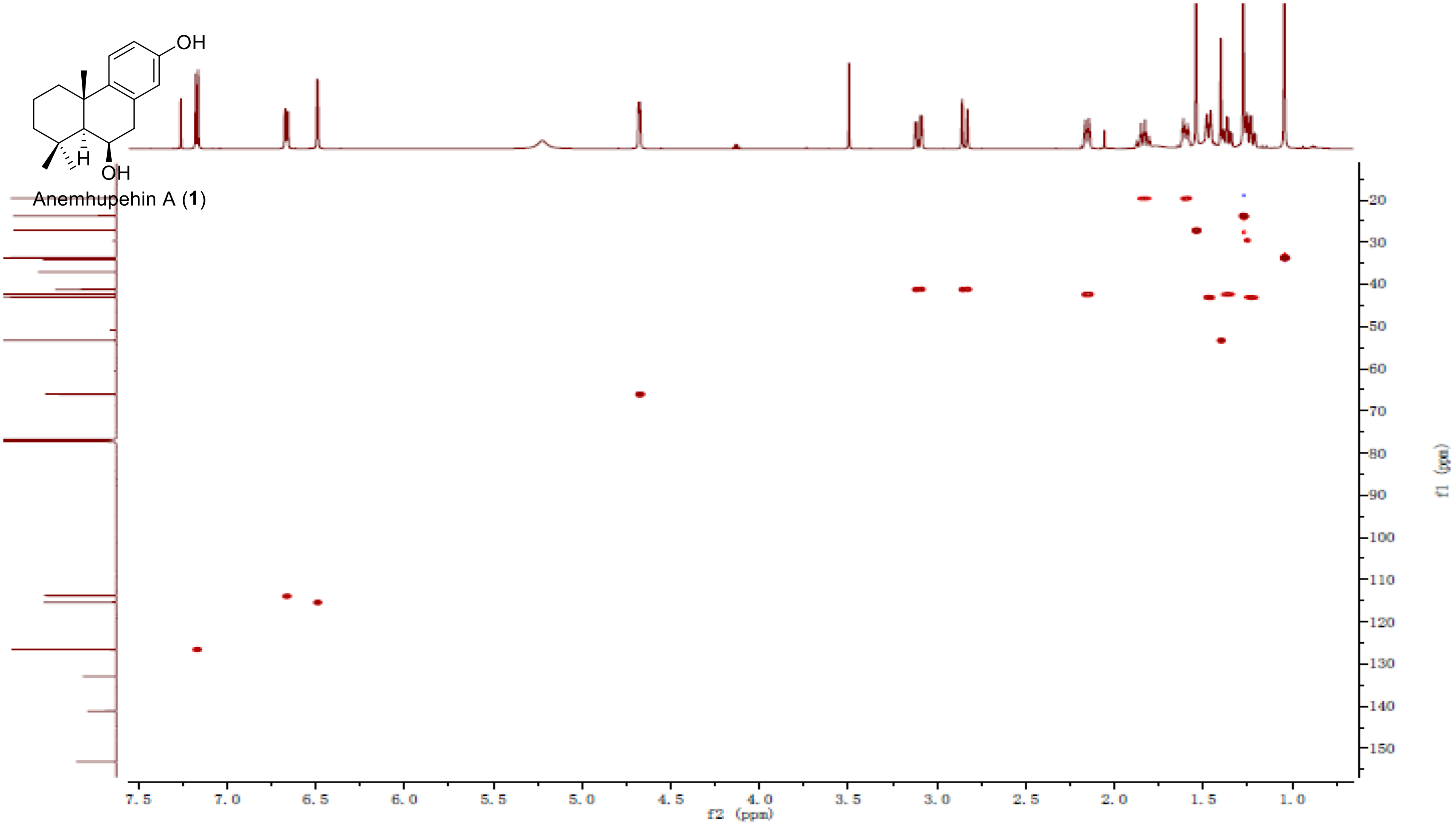

Figure S4. HMBC spectrum of anemhupehin A (**1**)

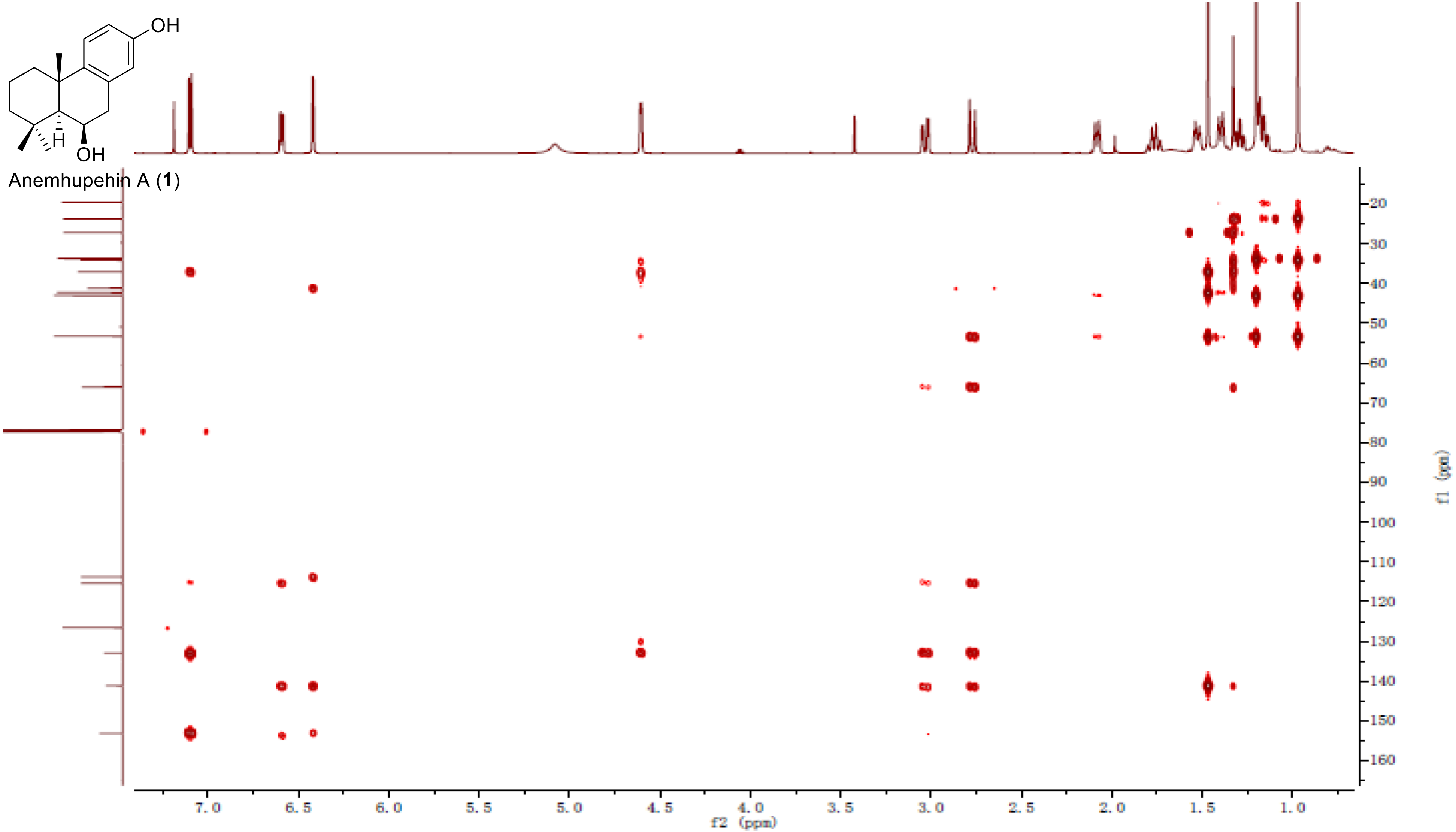

Anemhupehin A (**1**)

Figure S5. ^{1}H–^{1}H COSY spectrum of anemhupehin A (**1**)

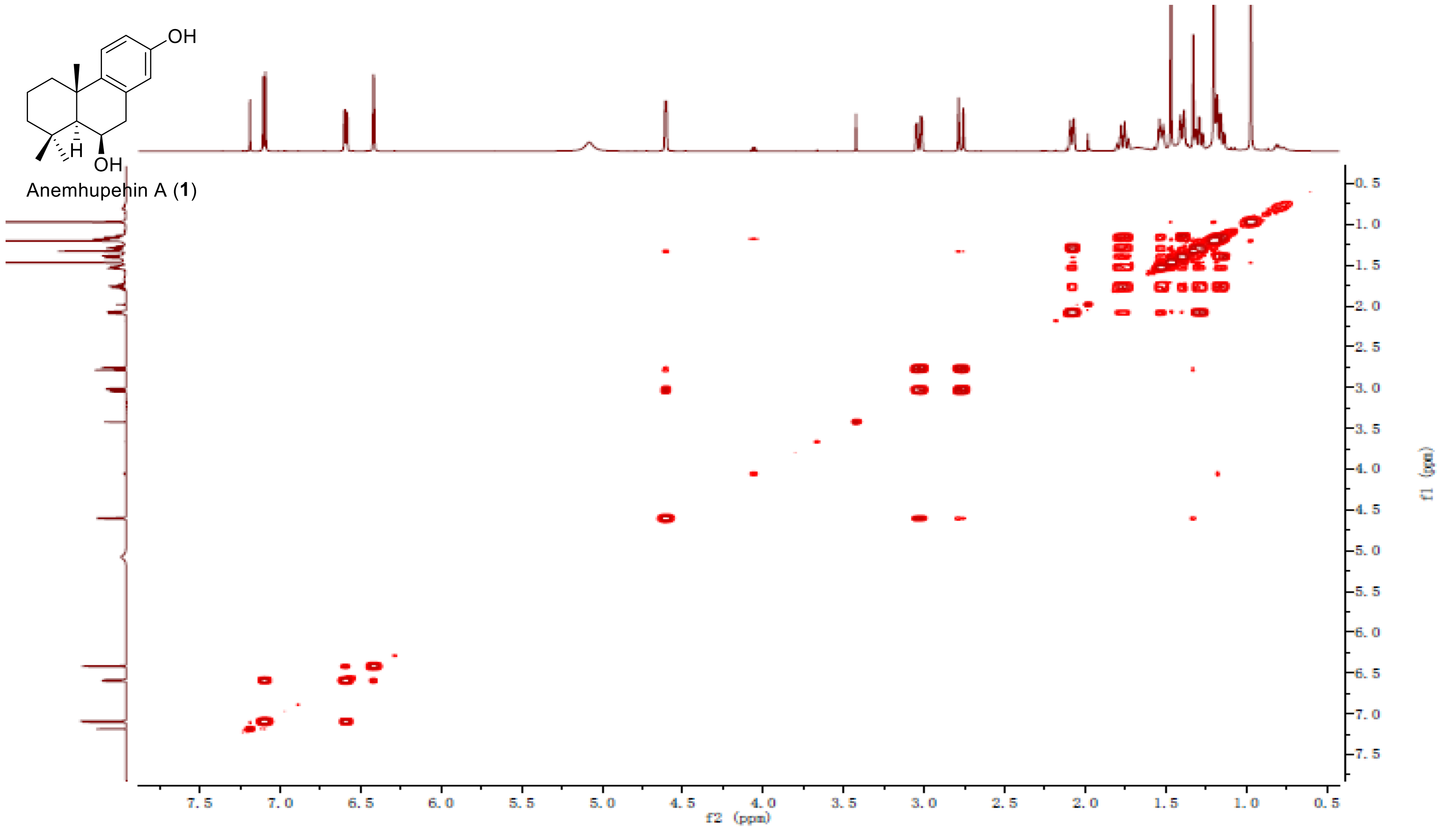

Figure S6. ROESY spectrum of anemhupehin A (**1**)

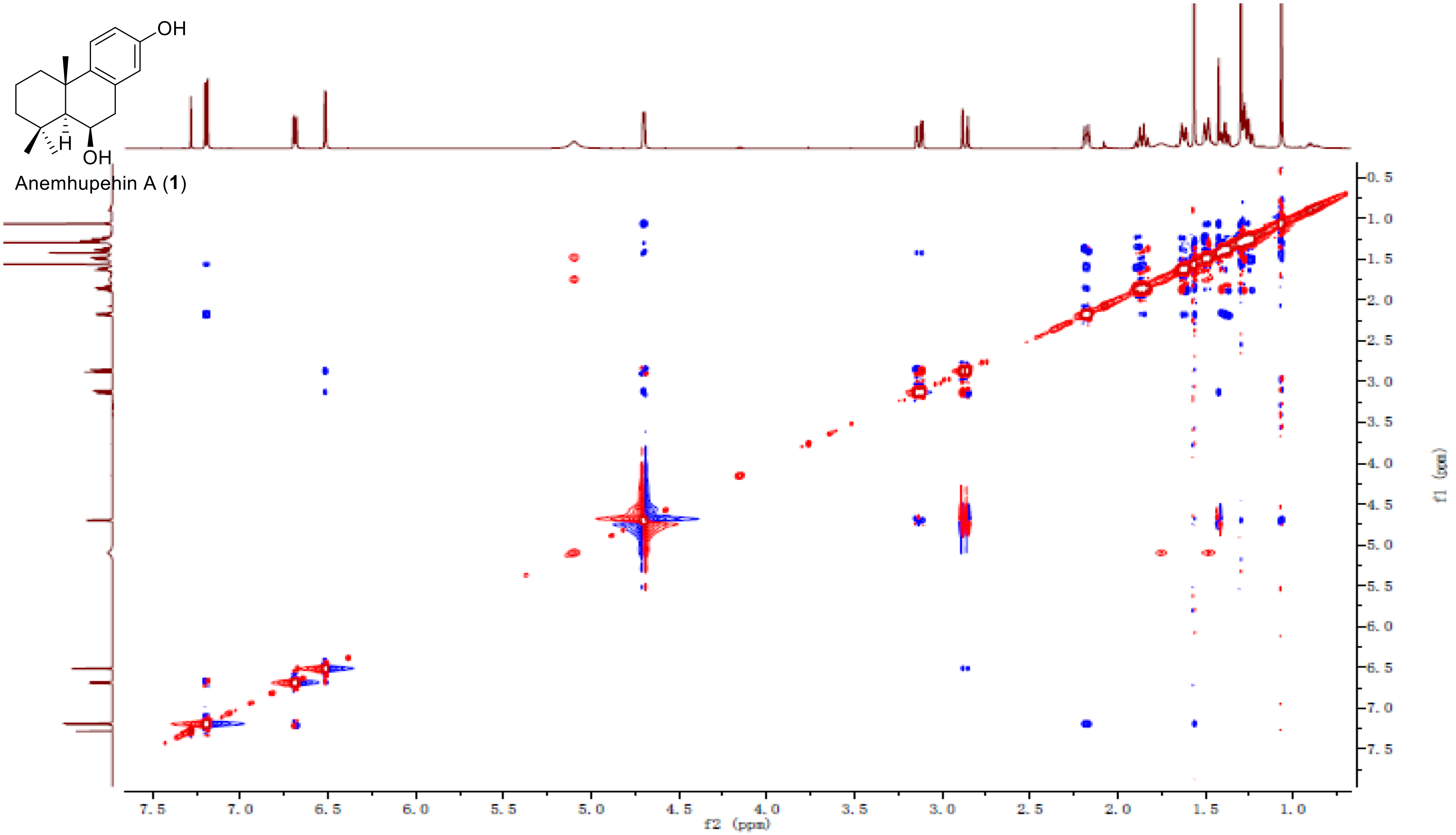

Figure S7. High resolution ESI mass spectrum of anemhupehin A (**1**)

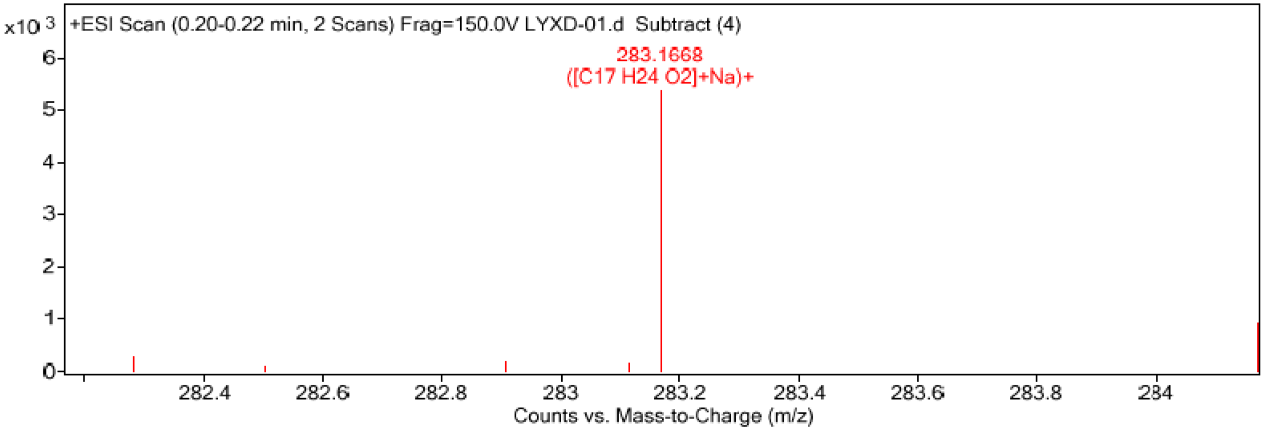

Peak List

m/z	z	Abund	Formula	Ion
97.9687		7619.4		
243.1741	1	12460.93		
255.1216	1	5588.78		
274.2738	1	29501.41		
283.1668	1	5391.83	C17 H24 O2	(M+Na)+
318.3	1	22544.3		
362.3256	1	5865.23		
453.1679	1	6994.53		

Formula Calculator Element Limits

Element	Min	Max
C	3	60
H	0	120
O	0	30
N	0	5

Formula Calculator Results

Formula	CalculatedMass	CalculatedMz	Mz	Diff. (mDa)	Diff. (ppm)	DBE
C17 H24 O2	260.1776	283.1669	283.1668	0.3	1.1	6.0000

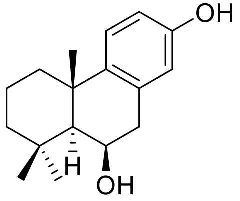

Anemhupehin A (**1**)

Figure S8. ^{1}H NMR spectrum of anemhupehin B (**2**)

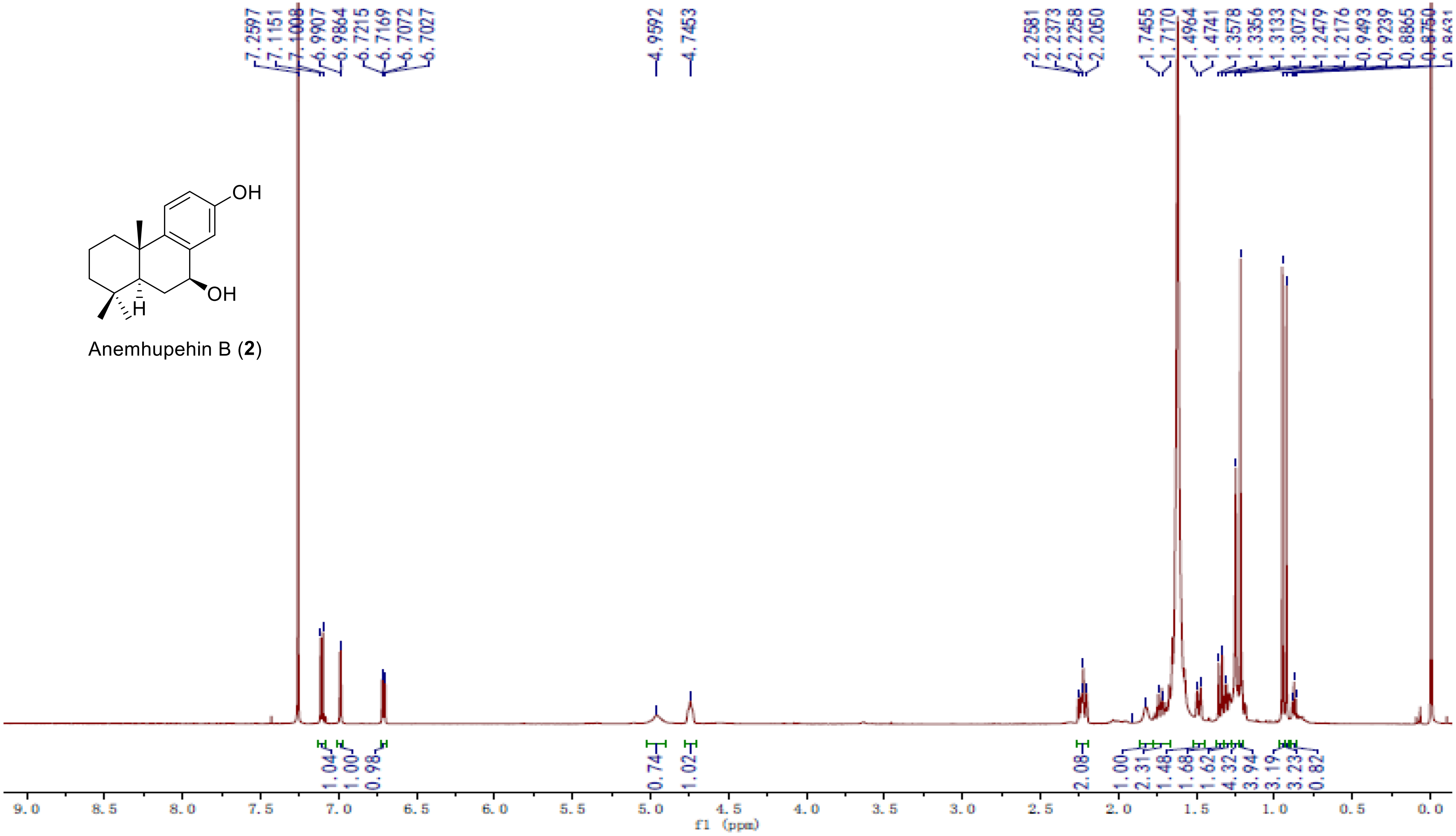

Figure S9. ^{13}C and DEPT NMR spectra of anemhupehin B (**2**)

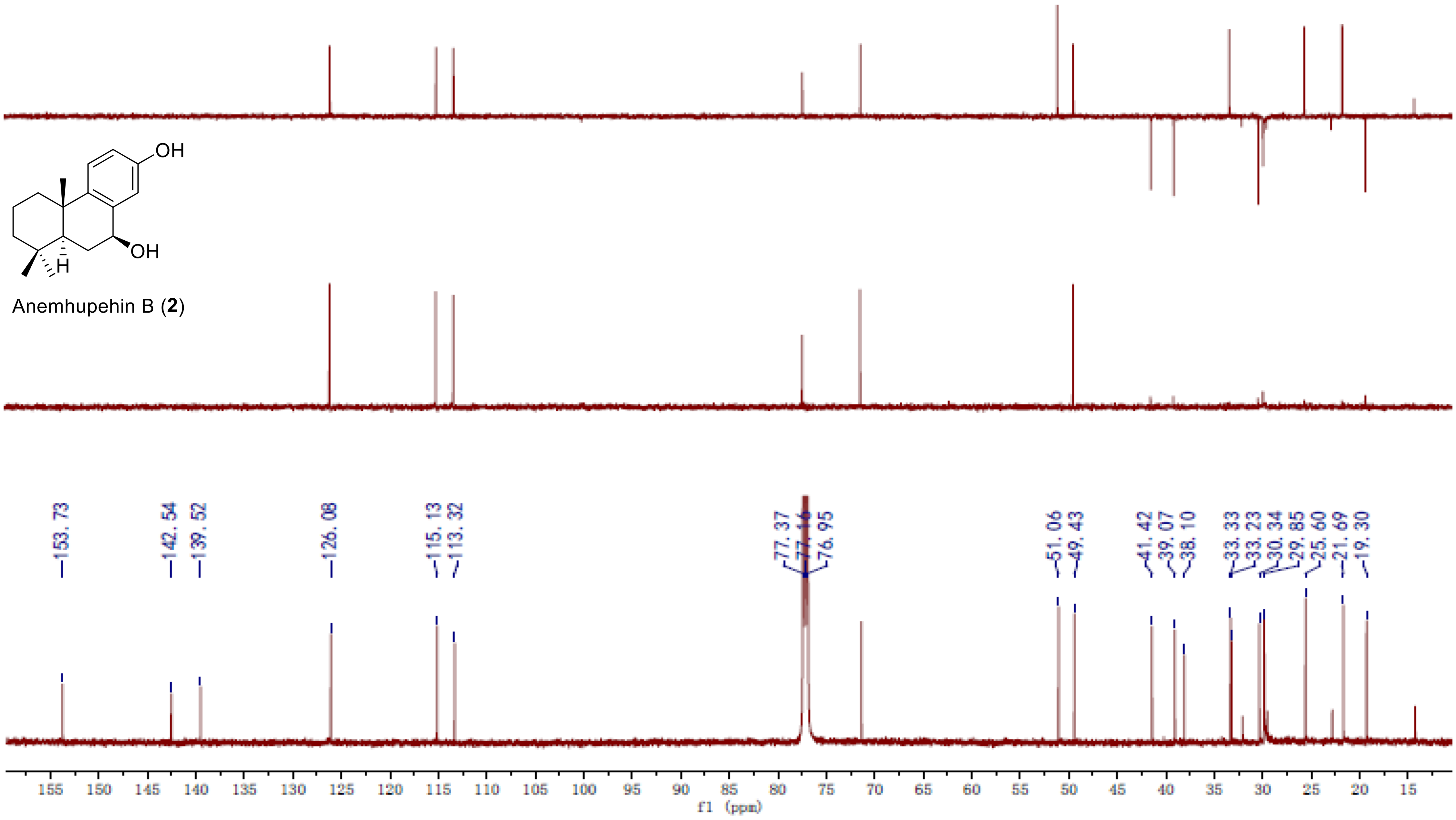

Figure S10. HSQC spectrum of anemhupehin B (**2**)

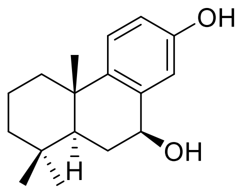

Anemhupehin B (**2**)

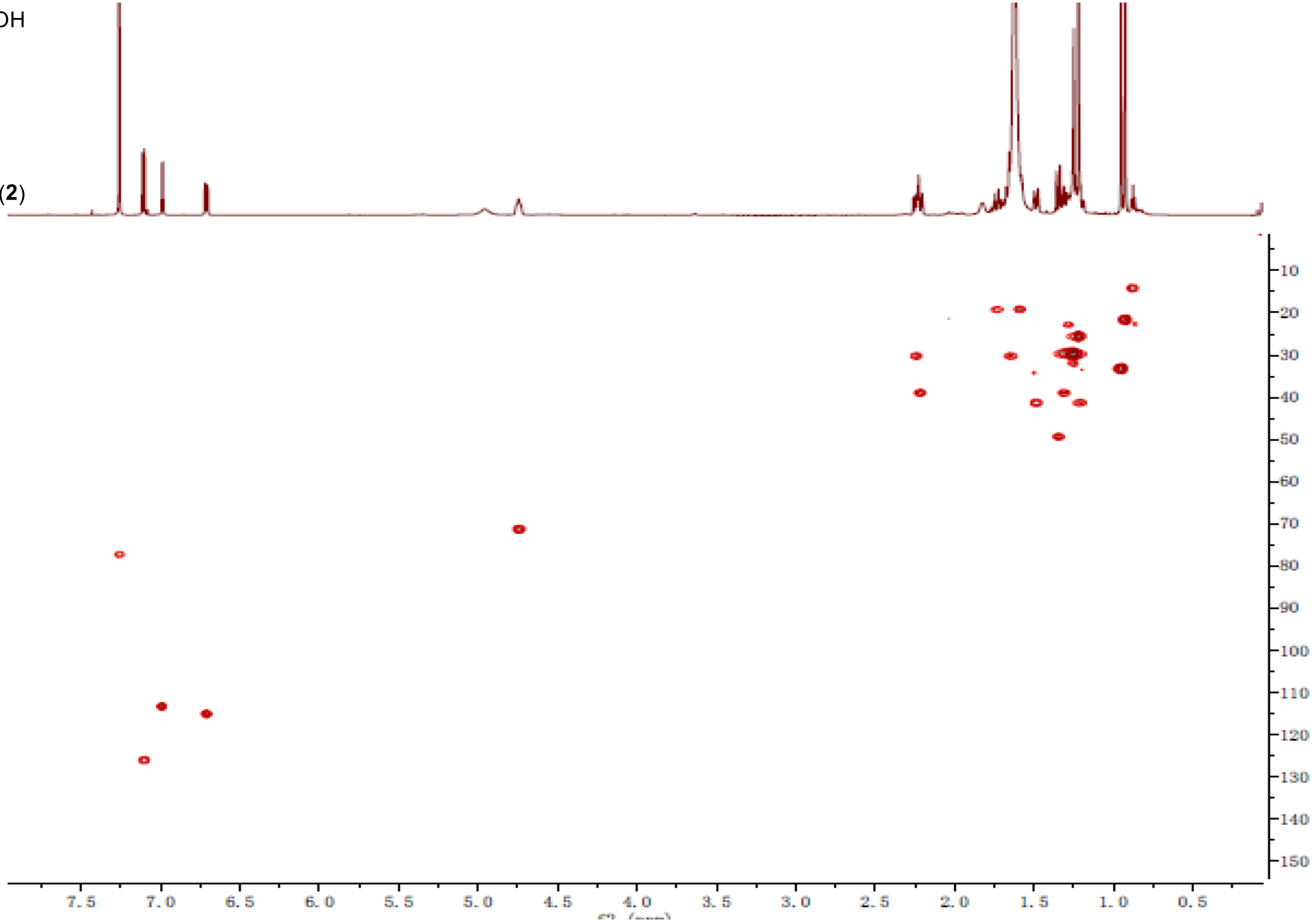

Figure S11. HMBC spectrum of anemhupehin B (**2**)

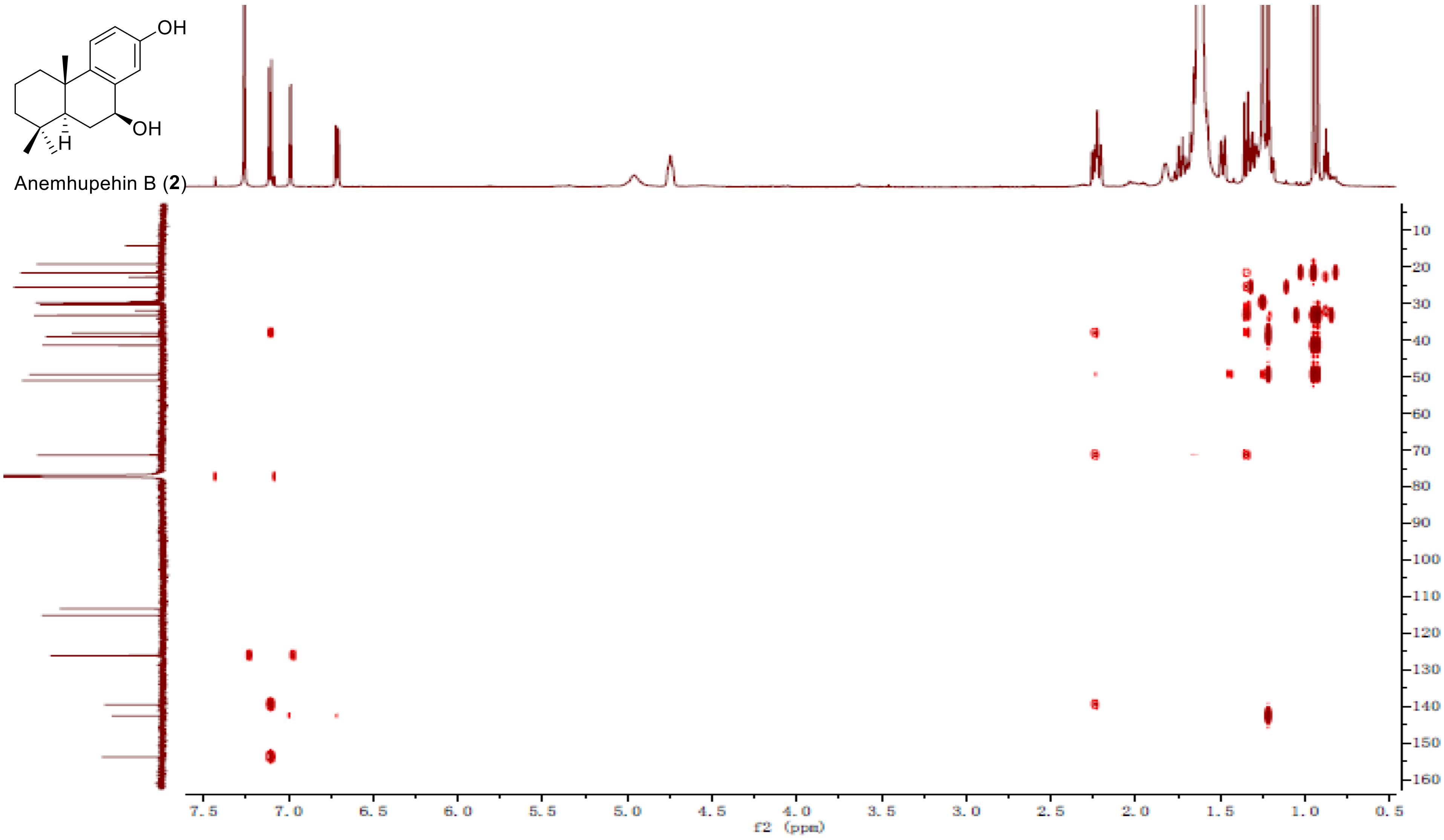

Figure S12. ^{1}H–^{1}H COSY spectrum of anemhupehin B (**2**)

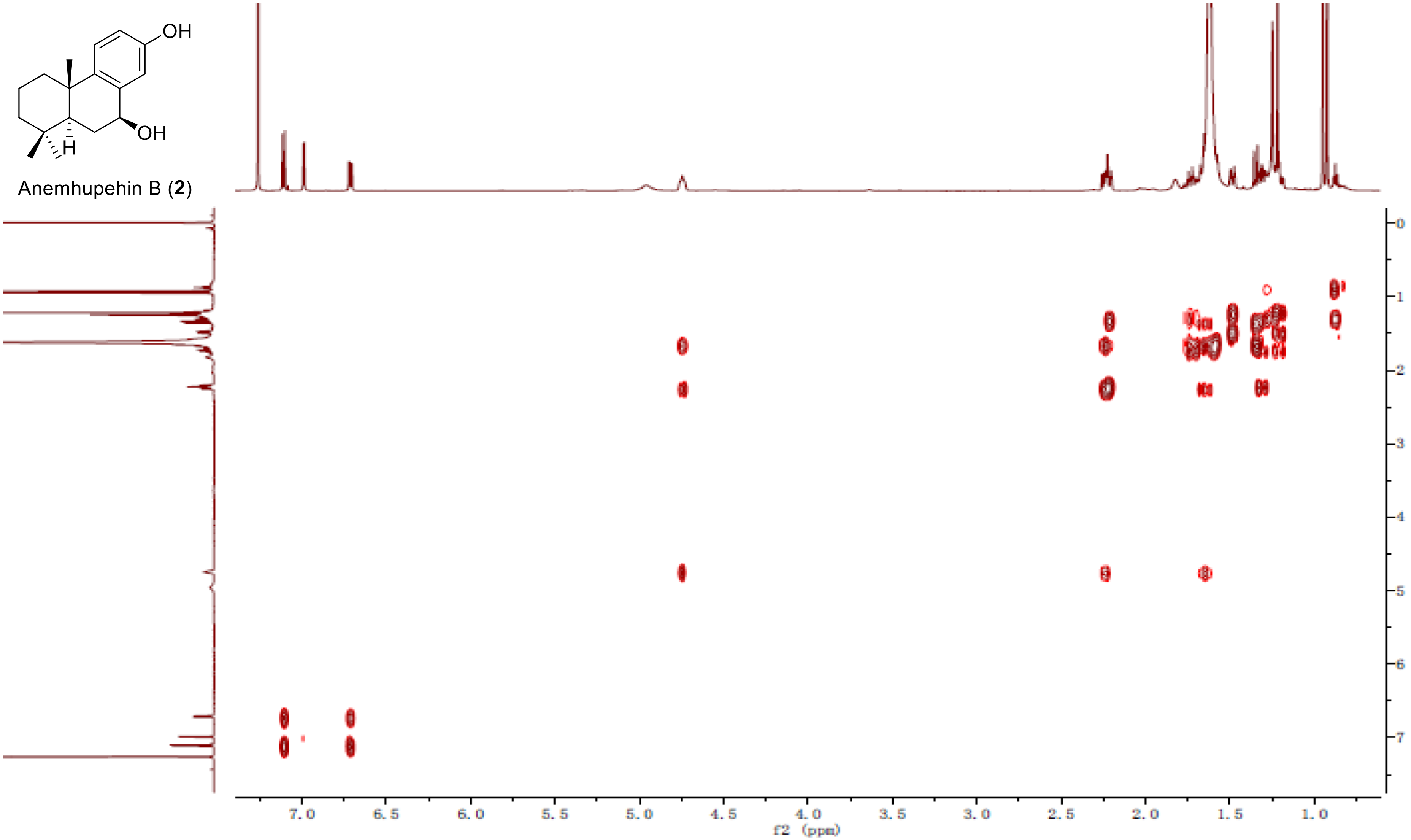

Figure S13. ROESY spectrum of anemhupehin B (**2**)

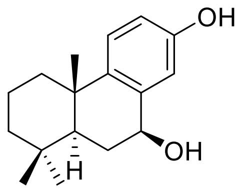

Anemhupehin B (**2**)

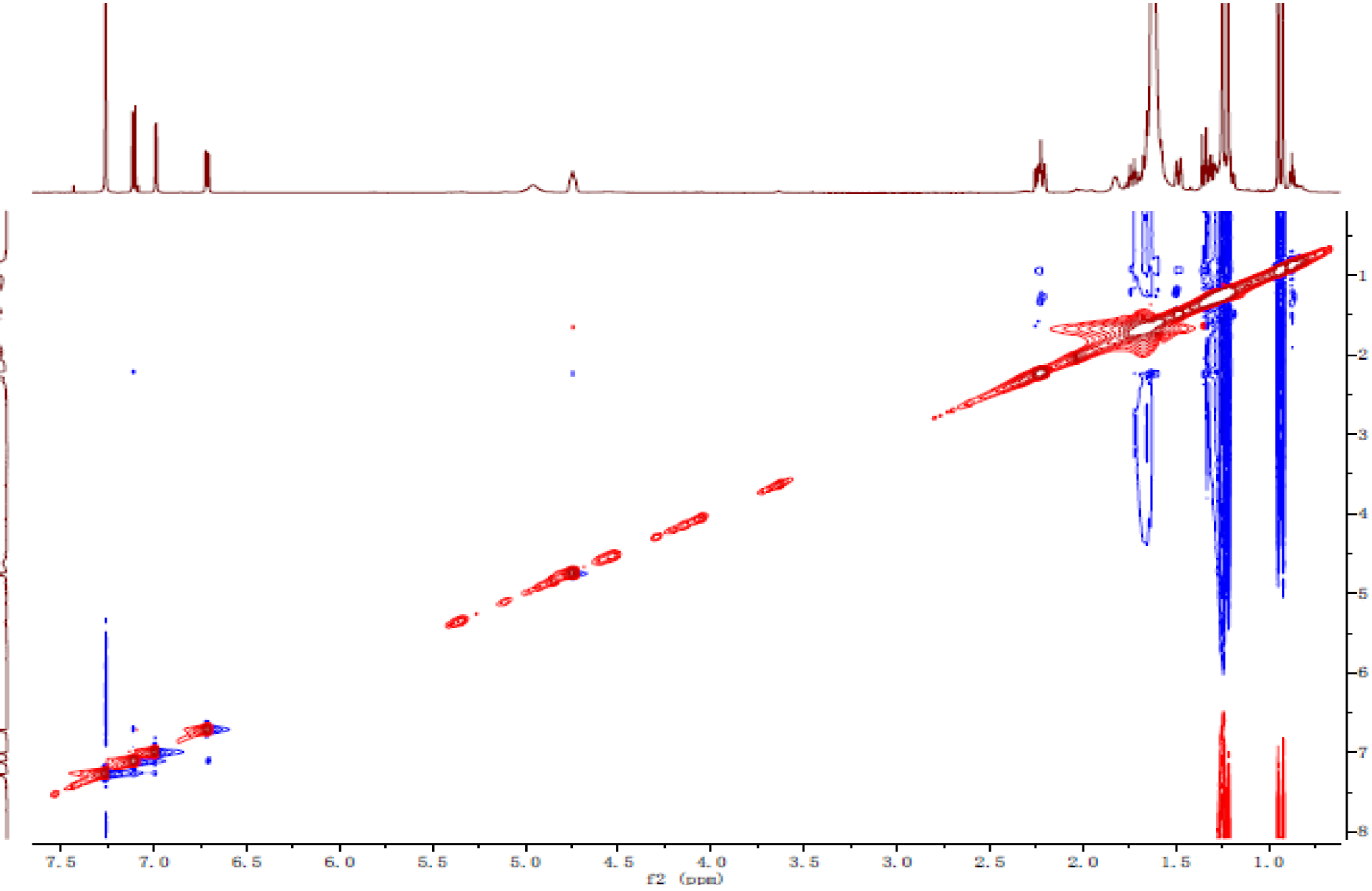

Figure S14. High resolution ESI mass spectrum of anemhupehin B (**2**)

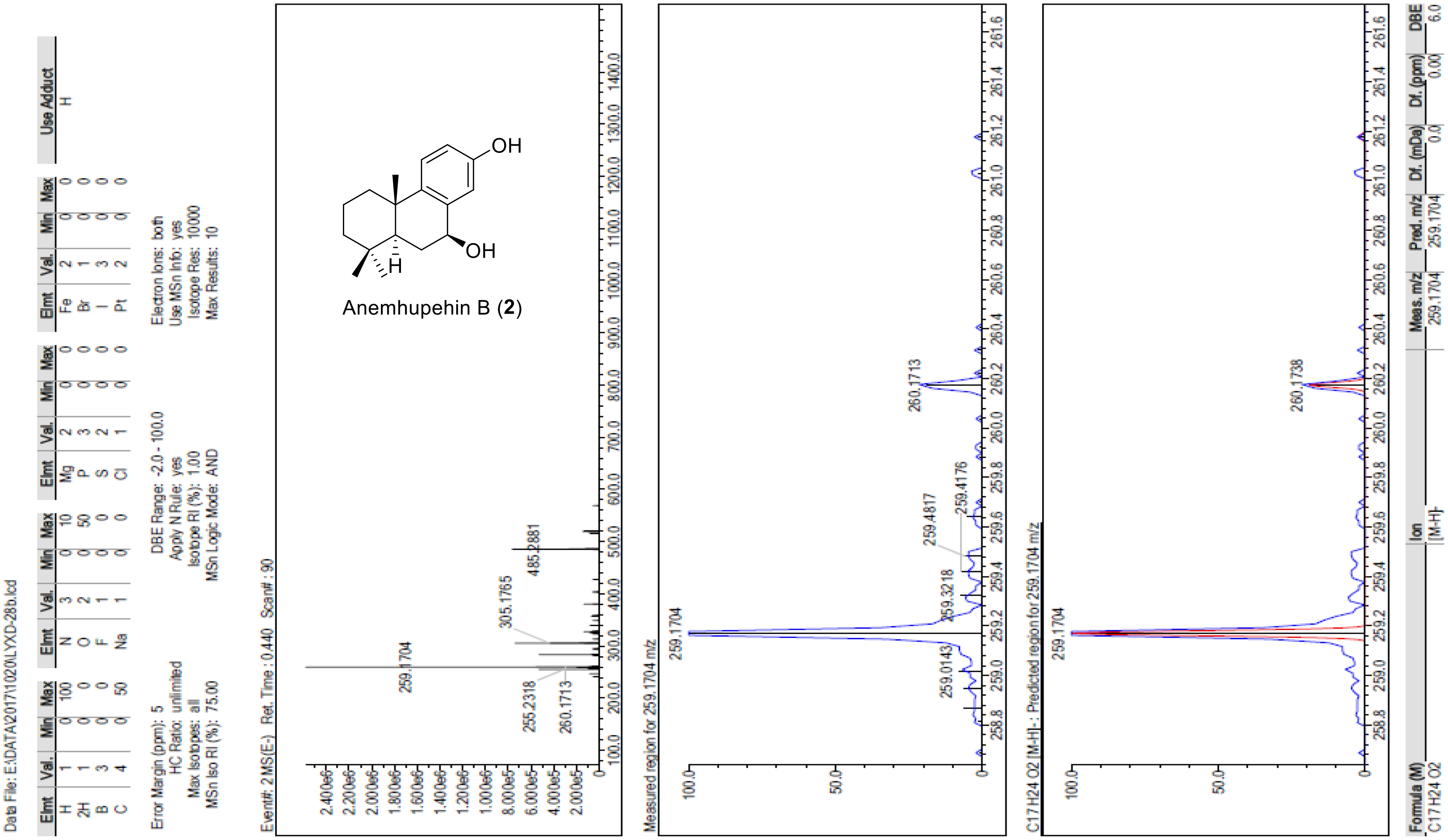

Figure S15. ^{1}H NMR spectrum of the mixture anemhupehins B + C (**2** + **3**)

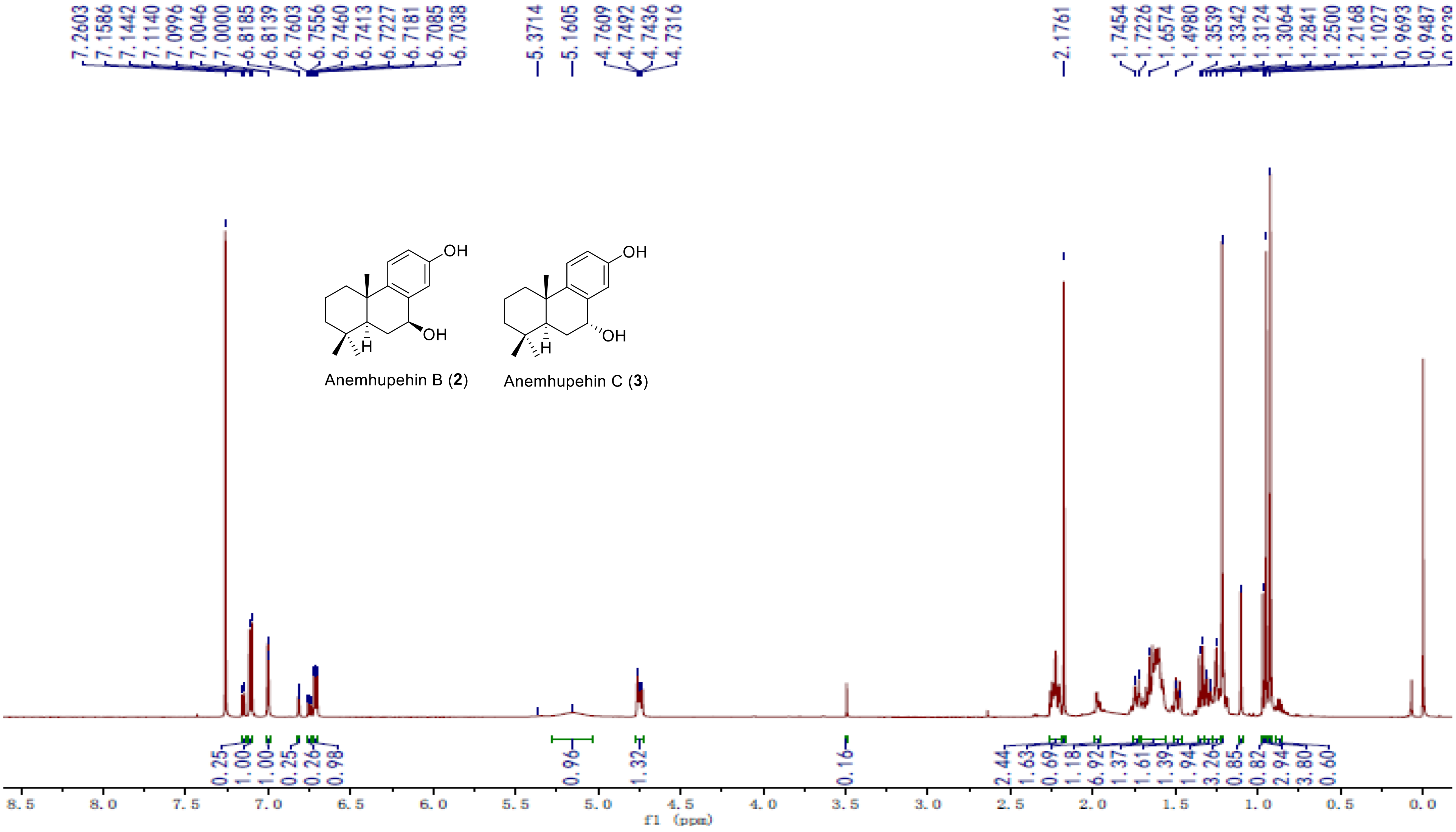

Figure S16. ^{13}C and DEPT NMR spectra of the mixture anemhupehins B + C (**2** + **3**)

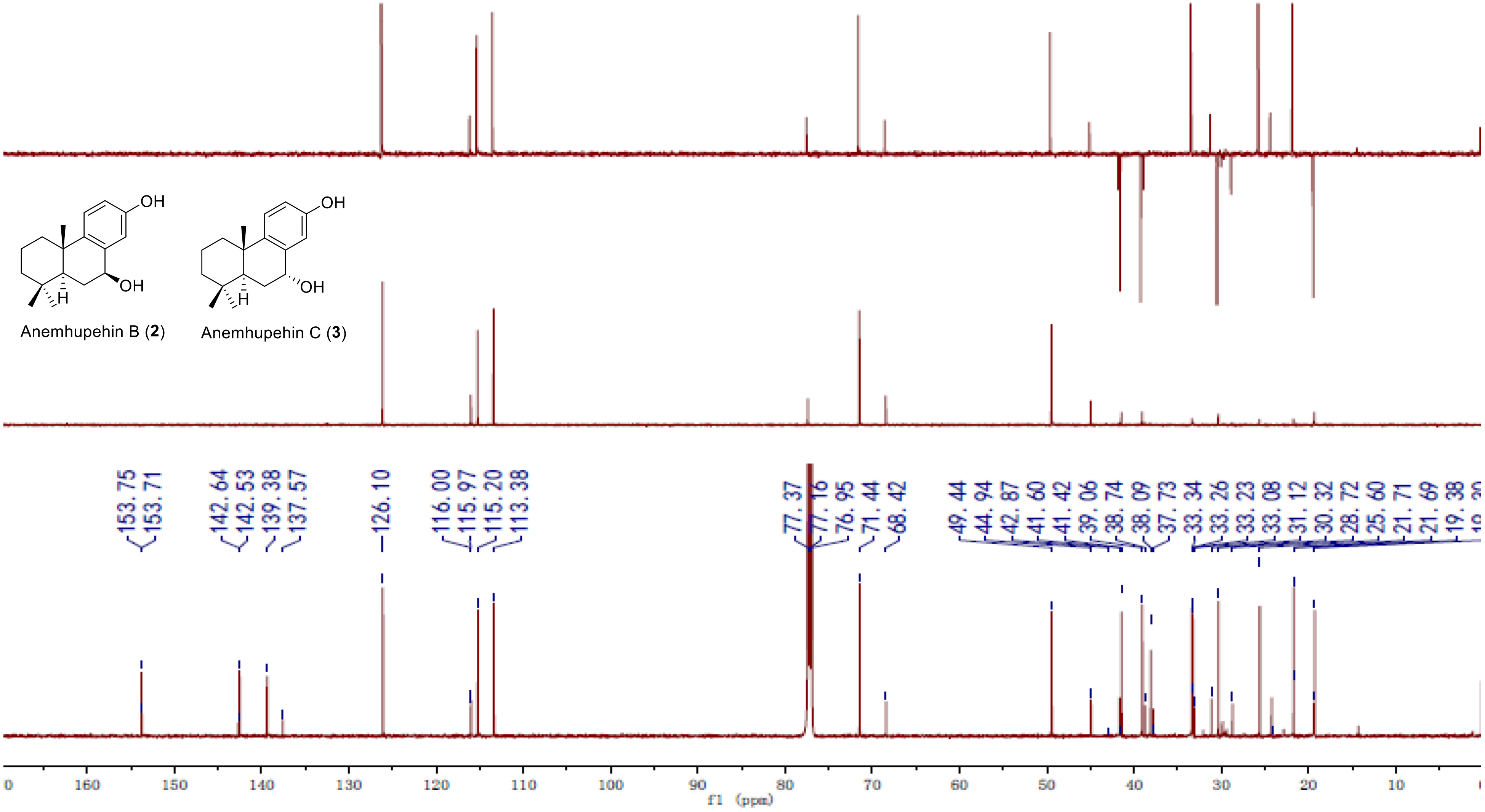

Figure S17. HSQC spectrum of the mixture anemhupehins B + C (**2** + **3**)

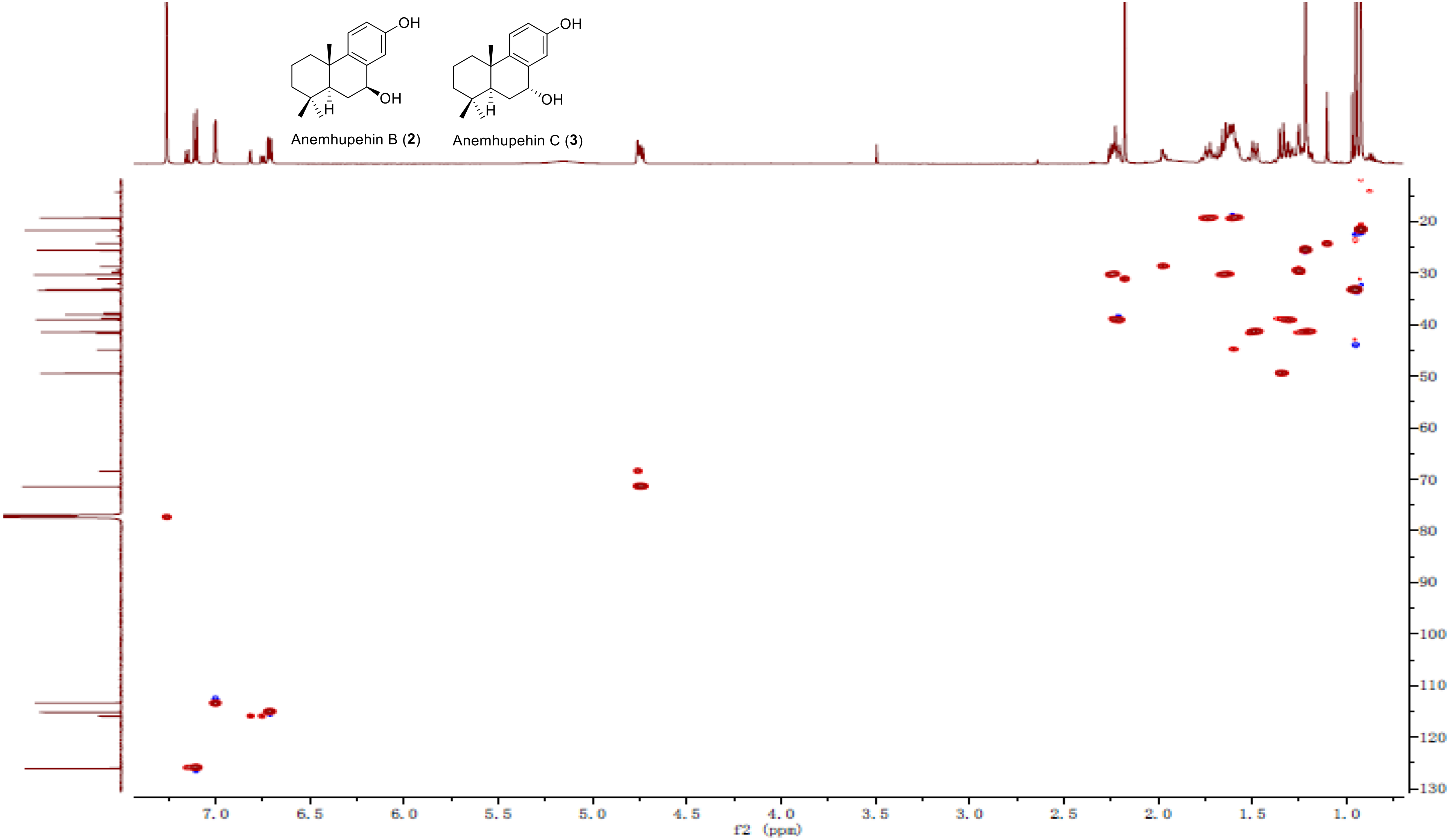

Figure S18. HMBC spectrum of the mixture anemhupehins B + C (**2** + **3**)

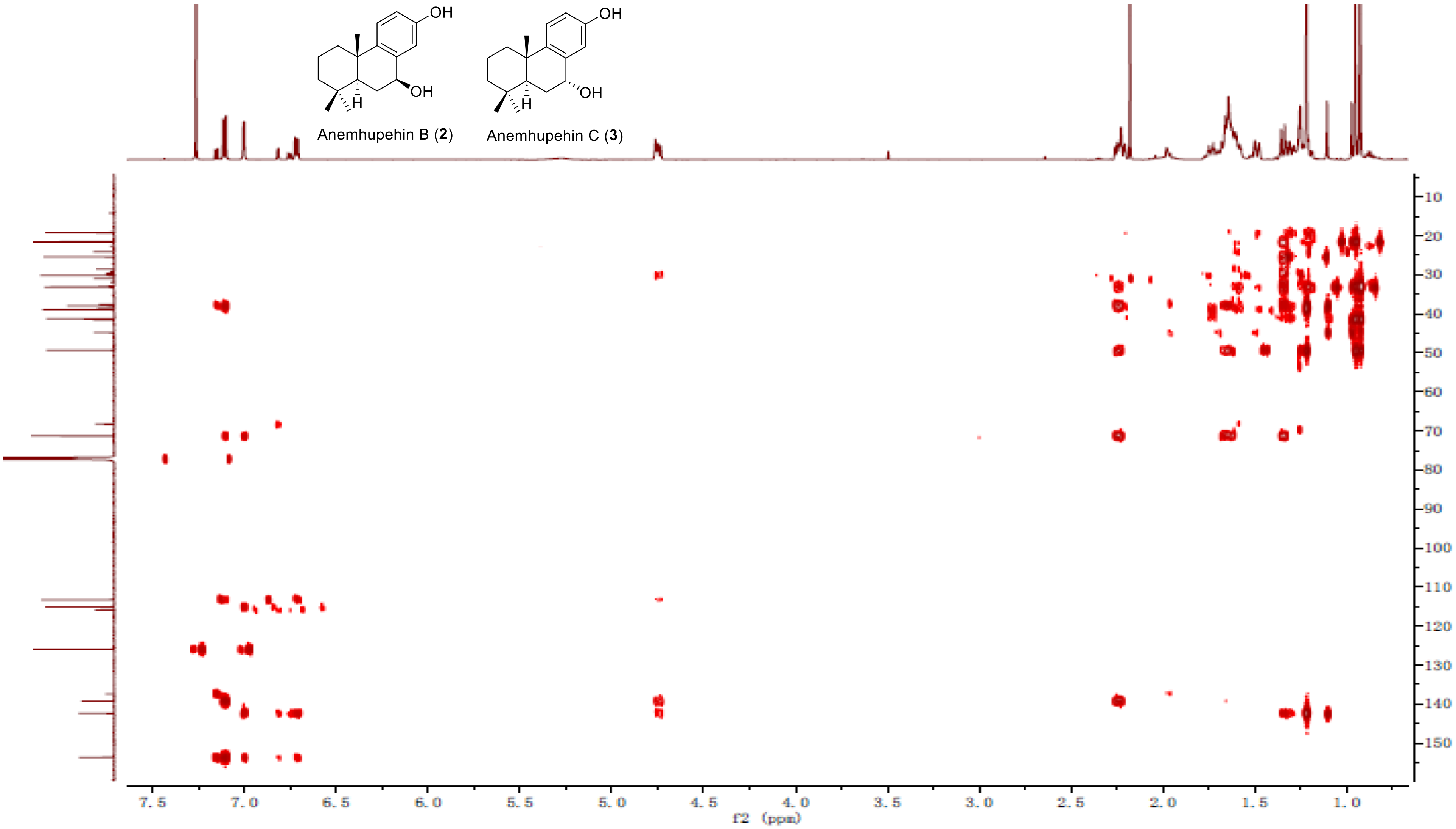

Figure S19. ^{1}H–^{1}H COSY spectrum of the mixture anemhupehin B + C (**2** + **3**)

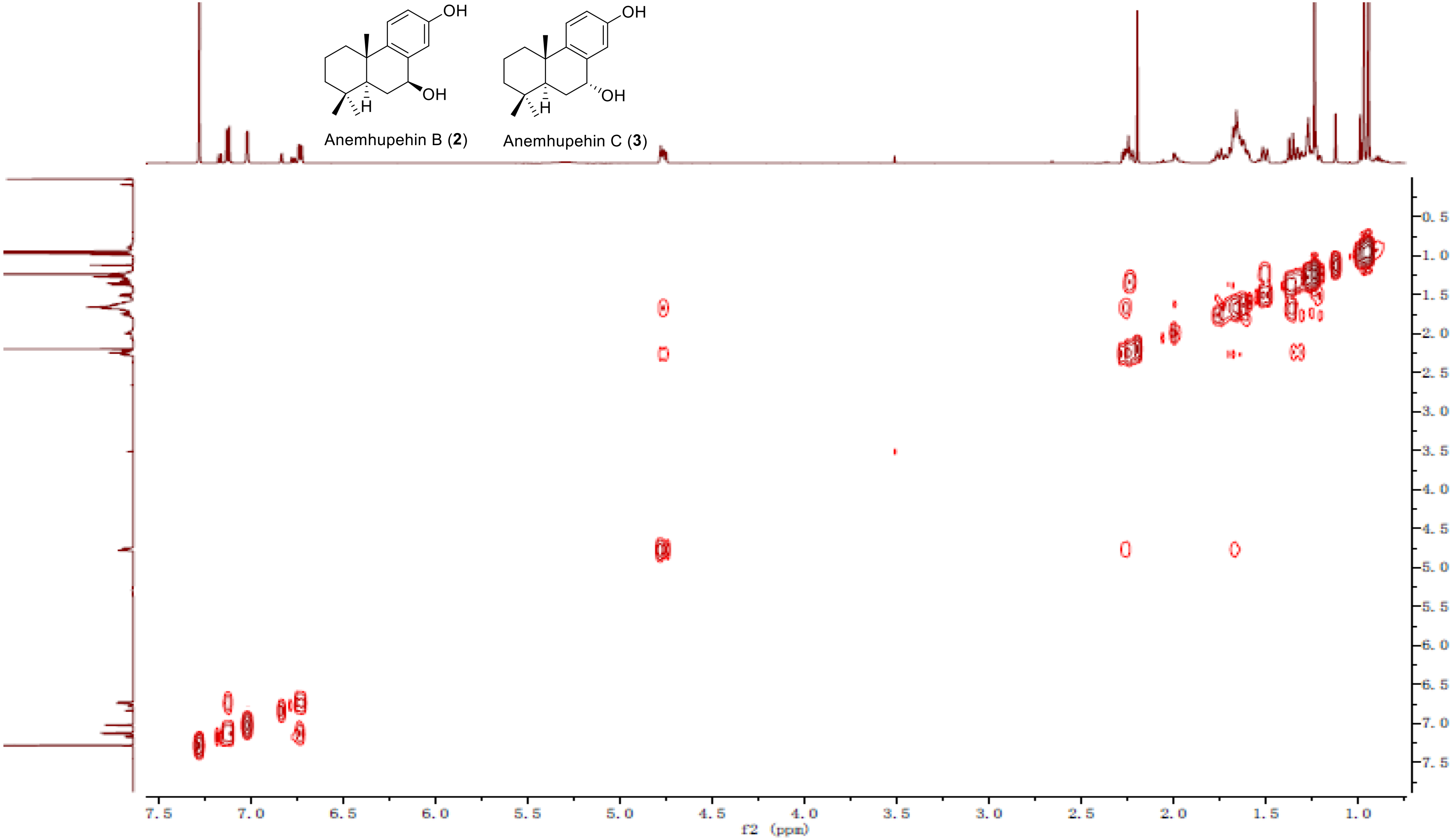

Figure S20. ROESY spectrum of the mixture anemhupehin B + C (**2** + **3**)

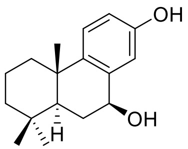

Anemhupehin B (**2**)

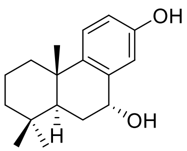

Anemhupehin C (**3**)

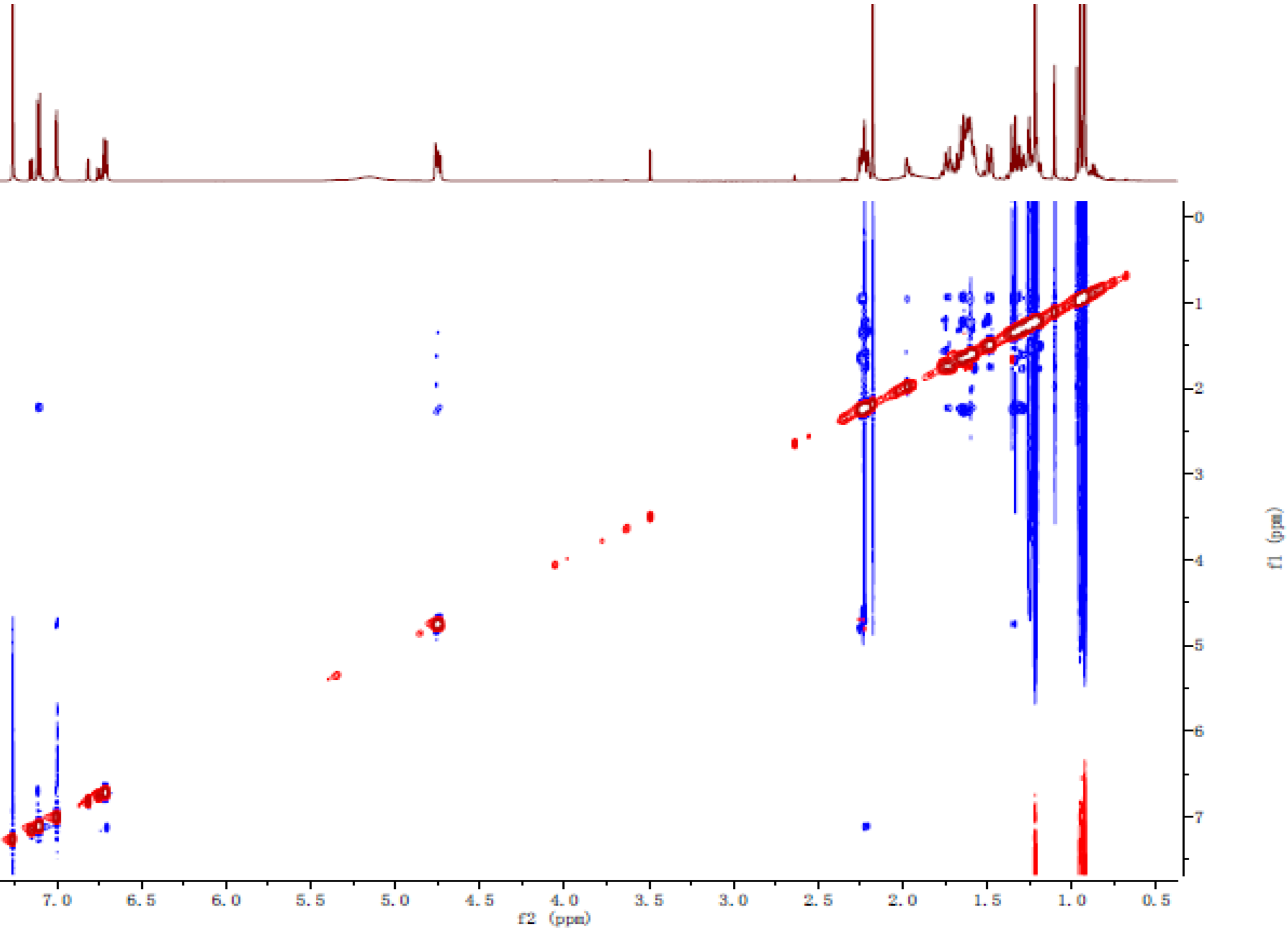

Figure S21. High resolution ESI mass spectrum of anemhupehin C (**3**)

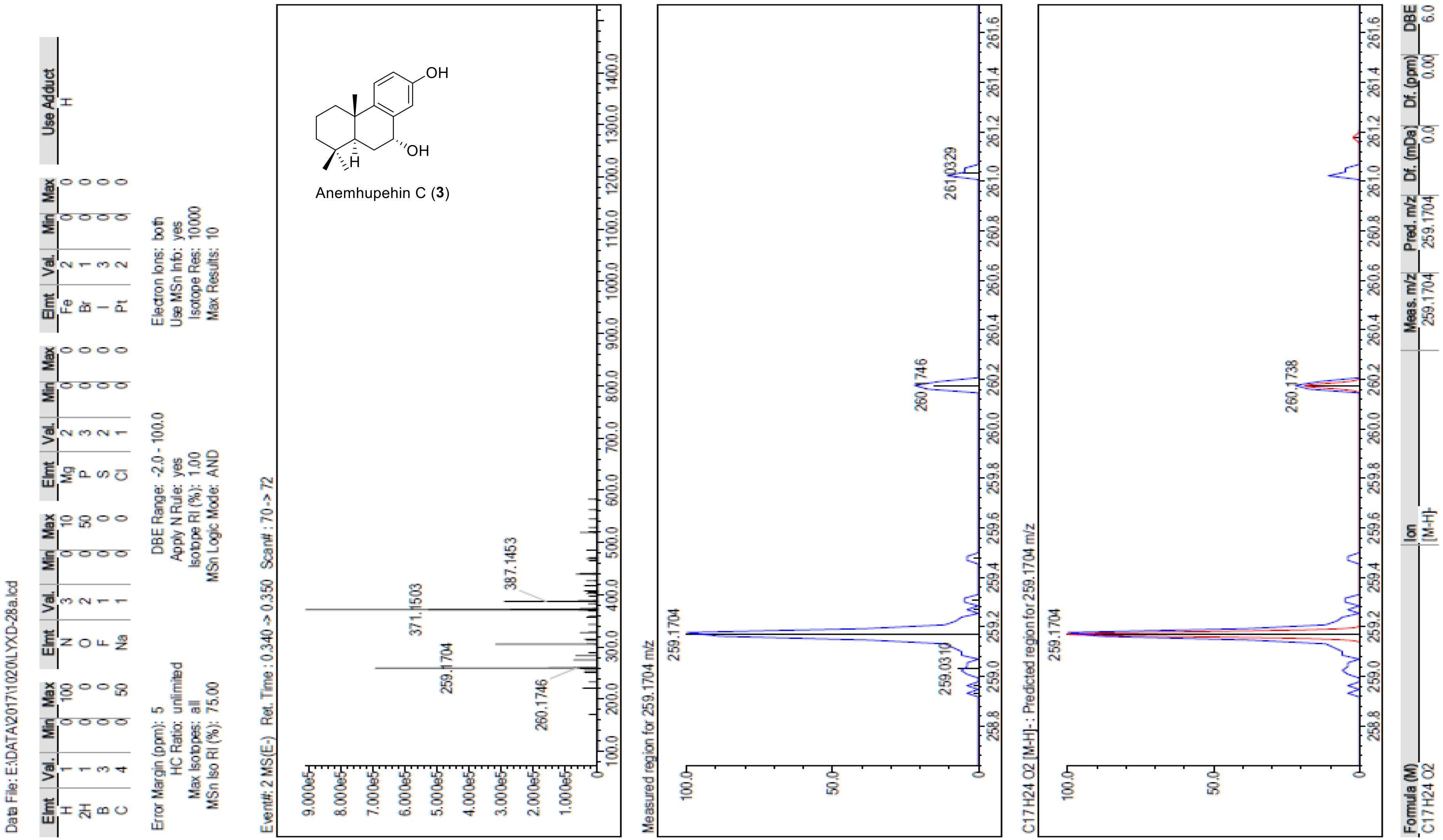

Supplement: Supplementary file 1 — Supplementary material 1 (PDF 1409 kb) [file 13659_2017_146_MOESM1_ESM.pdf]
